# Alpha-thalassaemia early eluting peak for alpha-thalassaemia --^SEA^ carrier screening: a multicentre diagnostic comparison with immunochromatographic strip test and haemoglobin H inclusion test

**DOI:** 10.1007/s00277-026-07102-0

**Published:** 2026-06-19

**Authors:** Wing Kit Lam, Carmen Michelle Yuen, Lawrence Lap Chi Tsui, Ting Hon Stanford Li, Vivian Ka Pik Yeung, Albert Chun Fung Sin, Tsz Fung Wong, Winnie Yim Fong Law, Christina Pui Ying Fan, Lok Nga Ko, Vivian Hoi Kei Woo, Kit Yu Chan, Tsz Ning Chan, Tze Wing Fan, Lok Han Too, Chi Keung Cheng, Man Ling Wong, Aves Hui Hsuan Wu, Benny Man Wai Lit, Yu Fong Wong, Man Wai Chan, Chun Him Ip, Julia Cheuk Ting Leung, Po Chun Wong, Kei Ching Yuen, Wang Ho Yuen, Hoi Ching Wong, Jamilla Wai Yan Li, Anskar Yu Hung Leung, Joyce Sin Cheung, Natalie Pui Ha Chan, Margaret Heung Ling Ng, Joyce Hoi Yi Kwong, Eudora Yu De Chow, Wai Shan Wong, Kate Fung Shan Leung, Sze Fai Yip

**Affiliations:** 1 G/F, Department of Clinical Pathology, Tuen Mun Hospital, 23 Tsing Chung Koon Road, Tuen Mun, Hong Kong, China; 2 Department of Medicine, Li Ka Shing Faculty of Medicine, The University of Hong Kong, Hong Kong, China; 3 Blood Cancer Cytogenetics and Genomics Laboratory, Department of Anatomical and Cellular Pathology, Princes of Wales Hospital, The Chinese University of Hong Kong, Hong Kong, China; 4 Department of Pathology, United Christian Hospital, Hong Kong, China; 5 Department of Pathology, Queen Elizabeth Hospital, Hong Kong, China; 6 Department of Pathology, Princess Margaret Hospital, Hong Kong, China; 7 Department of Pathology, Li Ka Shing Faculty of Medicine, The University of Hong Kong, Hong Kong, China; 8 Department of Anatomical and Cellular Pathology, Princes of Wales Hospital, Hong Kong, China; 9 Department of Pathology, Queen Mary Hospital, Hong Kong, China

**Keywords:** Alpha-thalassaemia, Immunochromatographic strip test, Haemoglobin H inclusion test, Alpha-thalassaemia early eluting peak

## Abstract

While high-performance liquid chromatography (HPLC) is well-established for β-thalassaemia and haemoglobinopathies, phenotypic screening for α^0^-thalassaemia has been limited. To address this limitation, we aimed to translate the discovery of the α-thalassemia early eluting peak (αEEP) in HPLC into clinical practice by comparing its diagnostic performance with other existing methods (haemoglobin H inclusion test [HbHi] and immunochromatographic strip test [ICT]) in a multicentre setting, and elucidating the nature of the αEEP by liquid chromatography-tandem mass spectrometry (LC-MS/MS). With a cohort of 820 genotyped patients, the αEEP showed superior diagnostic performance in detecting --^SEA^ (sensitivity 99.6%, specificity 100%) compared with HbHi (sensitivity 95.8%, *P* = 0.006; specificity 97.3%, *P* < 0.001) and ICT (sensitivity 95.8%, *P* = 0.006; specificity 75.4%, *P* < 0.001). Both HbHi and ICT showed reduced sensitivity in β-thalassaemia carriers versus non-carriers. ICT showed reduced specificity when Hb F ≥ 1% compared with < 1%. The αEEP remained robust across all subgroups. LC-MS/MS revealed a strong association between the αEEP and embryonic ζ-globin chains (*P* < 0.001). The αEEP offered cost reductions of 98.6% over HbHi and 97.3% over ICT. Collectively, the αEEP is a highly reliable and cost-effective marker for detecting --^SEA^ carriers, enabling a novel “all-in-one” HPLC screening strategy for --^SEA^, β-thalassaemia and haemoglobinopathies. Trial registration number: not applicable.

**Supplementary Information:**

The online version contains supplementary material available at https://doi.org/10.1007/s00277-026-07102-0.

## Introduction

Thalassaemia is one of the most common inherited disorders globally, causing quantitative imbalance of globin chains due to mutations in the α-, β- and δ-globin genes and consequently ineffective erythropoiesis [1]. The α- and β-thalassaemia are particularly prevalent in regions such as Southeast Asia, resulting in significant public health burden [2]. The global prevalence of α- and β-thalassaemia is approximately 5% and 1.5%, respectively [3, 4]. Clinically significant thalassaemia can develop in offspring if both parents are carriers, including β-thalassaemia intermedia or major (both functional β-globin genes lost), haemoglobin (Hb) H disease (three α-globin genes lost), and the fatal Hb Bart’s hydrops foetalis (all four α-globin genes lost).

The α^0^-thalassaemia carrier screening is critical especially in high-prevalence areas. The Southeast Asian type deletion (--^SEA^) is the most prevalent mutation in Southeast Asia and the West Pacific region [5–8]. While β-thalassaemia and other haemoglobinopathies are readily detected by high-performance liquid chromatography (HPLC) or capillary electrophoresis, only red cell indices have been recommended for triaging patients for α^0^-thalassaemia genotyping [9, 10]. Although genotyping remains the gold standard, a reliable and cost-effective phenotypic screening for high-prevalence areas is essential, as red cell indices in iron deficiency can mimic those seen in thalassaemia [11, 12].

Microscopy-based Hb H inclusion test (HbHi) detecting Hb H inclusions (β_4_ tetramer) is still widely used but is labour-intensive, not standardised and has limited sensitivity [13, 14]. In the last decade, an immunochromatographic strip test (ICT) based on the detection of Hb Bart’s (γ_4_ tetramer) has been developed, with a reported sensitivity of 97.4% [15, 16].

**Fig. 1:**
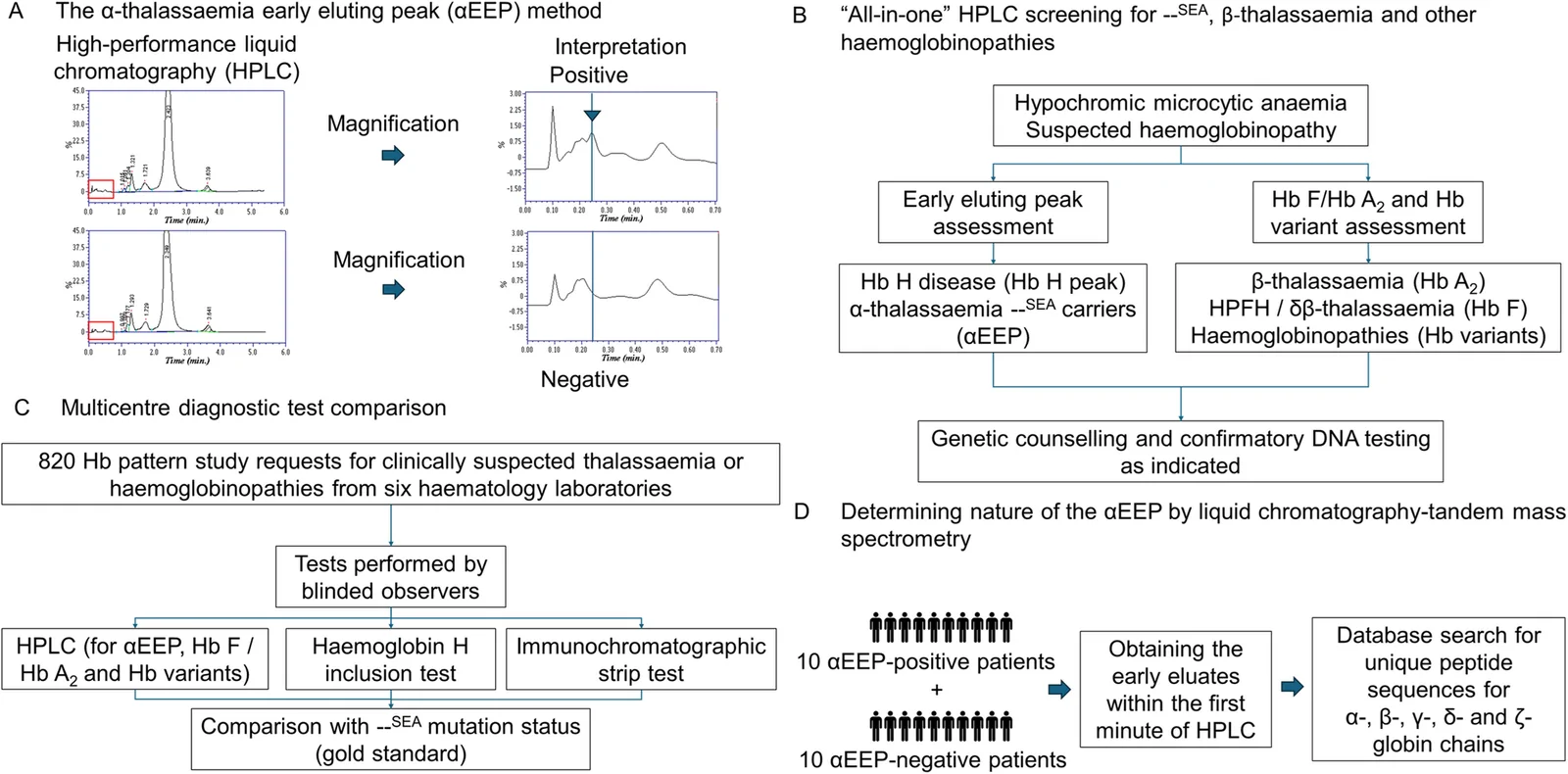
(**A**) The α-thalassaemia early eluting peak (αEEP) assessment involves magnifying the area of interest (red box) in the chromatogram of high-performance liquid chromatography (HPLC). The αEEP appears at a retention time of about 0.24 min, and is interpreted as either ‘positive’ (arrowhead, upper panel) or ‘negative’ (lower panel). (**B**) The novel “all-in-one” strategy in thalassaemia and haemoglobinopathy screening combines standard haemoglobin analysis (Hb F, Hb A_2_, Hb variants and Hb H peak) by HPLC with an additional early eluting peak assessment for the αEEP. Results guide further genetic counselling and confirmatory DNA testing as indicated. (**C**) 820 patients were included in this multicentre diagnostic test comparison for blinded assessment of the αEEP, Hb H inclusion test and the immunochromatographic strip test. The results were compared against genotyping as the gold standard. (**D**) Early eluates from twenty patients (ten αEEP-positive and ten αEEP-negative) were analysed by liquid chromatography-tandem mass spectrometry to characterise the substance underlying the αEEP

More recently, the α-thalassaemia early eluting peak (αEEP) has emerged as a potential screening method for --^SEA^ carriers by utilising the mostly-unanalysed early eluting peak pattern in HPLC (Fig. 1A) [17, 18]. We hypothesised that the αEEP had better diagnostic performance compared with existing methods (i.e. ICT and HbHi), and was a biochemically sound marker for diagnostic use. This study endeavours to translate the discovery of the αEEP into clinical practice by conducting a multicentre, assessor blinded, head-to-head diagnostic comparison of the αEEP with HbHi and ICT, and to characterise the nature of the αEEP. Ultimately, we aimed to enable a novel “all-in-one” HPLC screening strategy for --^SEA^ carriers, β-thalassaemia and other haemoglobinopathies (Fig. 1B).

## Materials and methods

This multicentre, retrospective diagnostic test evaluation consisted of two major parts (Fig. 1C, D). The first was a multicentre diagnostic comparison of the αEEP, HbHi and ICT conducted at six tertiary referral hospitals in Hong Kong (Tuen Mun Hospital, Queen Mary Hospital, Prince of Wales Hospital, United Christian Hospital, Queen Elizabeth Hospital and Princess Margaret Hospital) providing regional haematology laboratory service with a catchment population of about 6.8 million (90.5% of the population) [19]. The Hb pattern study requests were received for investigation of anaemia, clinically suspected thalassaemia or haemoglobinopathies in daily practice. The second was a liquid chromatography-tandem mass spectrometry (LC-MS/MS) study to investigate the biochemical nature of the αEEP, performed by Tuen Mun Hospital and the Proteomics and Metabolomics Core Facility, Centre for PanorOmic Sciences, Li Ka Shing Faculty of Medicine, the University of Hong Kong. The study was performed in accordance with the Declaration of Helsinki, with ethics approval obtained for all participating centres after review by the Hospital Authority Central Institutional Review Board (Ref. CIRB-2023-174-1), Institutional Review Board of the University of Hong Kong / Hospital Authority Hong Kong West Cluster (Ref. UW 24–098), and The Joint Chinese University of Hong Kong (CUHK) - New Territories East Cluster Clinical Research Ethics Committee (Ref. 2024.113).

The diagnostic comparison was conducted across the six participating hospitals from March to December 2024. Inclusion criteria were: (1) ethylenediaminetetraacetic acid (EDTA)-anticoagulated peripheral blood sample received for Hb pattern study; (2) age ≥ 1 year; and (3) sufficient to perform all the tests: αEEP, ICT, HbHi and α-globin genotyping for common deletional and non-deletional mutations in Chinese (--^SEA^, 3.7 kb deletion [-α^3.7^], 4.2 kb deletion [-α^4.2^], Hb Constant Spring, Hb Quong Sze and codon 30 mutation), as described previously [17, 18]. Patients with Hb H disease or a history of blood transfusion within three months were excluded. Extended genotyping for Filipino (--^FIL^), Thai (--^THAI^) and Mediterranean (--^MED^) deletions modified from Tan et al. [20] was performed for non-Chinese patients with a negative initial α-globin genotyping result. The αEEP (from Variant II HPLC, Bio-Rad Laboratories, California, USA), a commercial ICT kit (i + Med Laboratories, Thailand), and in-house HbHi were assessed by trained, blinded observers (Fig. 1C). Detailed methods including materials, sample preparation, detection and interpretation are provided in the Supplementary Information.

For LC-MS/MS analysis, 10 mL of first-minute HPLC eluates from each of the twenty peripheral EDTA blood samples (ten αEEP-positive, ten αEEP-negative) were collected (Fig. 1D). Unique peptide sequences for haemoglobin subunits were identified via database search during software analysis. Further details are provided in the Supplementary Information.

### Statistical analysis

Sample size was determined assuming a 5% discordance rate between ICT or HbHi and αEEP results. A sample size of 700 provided 85% power to detect differences at a two-sided type I error rate of 5%.

Statistical analyses were performed using GraphPad Prism version 10.4.2 (GraphPad Software, California, USA) and SPSS Statistics version 30.0 (IBM Corporation, New York, USA). Equivocal αEEP results were considered negative in the calculation of diagnostic performance due to potential inter-observer variability. The test performance was primarily assessed for --^SEA^ detection based on sensitivity, specificity, positive predictive value (PPV) and negative predictive value (NPV) with 95% confidence intervals (95% CI) derived by the Wilson-Brown method. Differences in sensitivity and specificity between αEEP and HbHi or ICT for detecting --^SEA^ were assessed by McNemar’s test. Subgroup analyses compared test performance in detecting --^SEA^ according to the β-thalassaemia status and Hb F levels (< 1% versus ≥ 1%). Fisher’s exact test was used to evaluate associations between subgroups and test performance, and between the αEEP result and globin chain peptides detected by LC-MS/MS. All hypothesis tests were two-sided, and *P* values of < 0.05 were considered statistically significant. Costs for αEEP, HbHi and ICT were compared based on total manpower and reagent/consumable costs per 100 tests.

## Results

### Multicentre diagnostic comparison

#### Patient characteristics

**Fig. 2:**
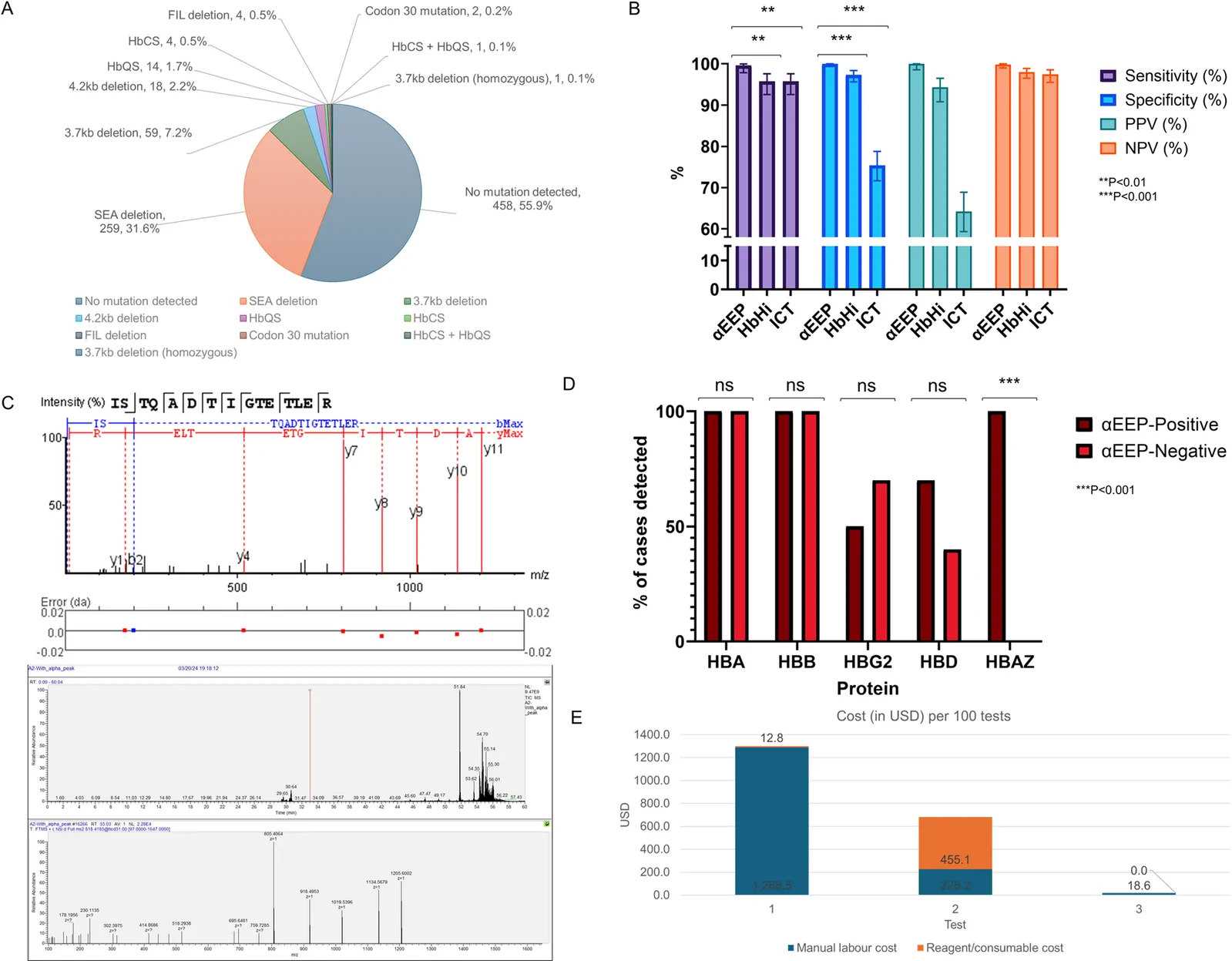
(**A**) The α-globin genotypes of patients included in the multicentre diagnostic test comparison. The Southeast Asian type deletion was the predominant mutation in this cohort (31.6% of patients). (**B**) The α-thalassaemia early eluting peak (αEEP) showed significantly higher sensitivity and specificity compared with haemoglobin H inclusion test (HbHi) and immunochromatographic strip test (ICT). (**C**) Liquid chromatography-tandem mass spectrometry (LC-MS/MS) results from an αEEP-positive patient. The unique peptide sequence of the ζ-globin chain (ISTQADTIGTETLER) was detected in the tandem mass spectrometry (upper panel), and the corresponding peak (red) appeared at 33.03 min in liquid chromatography (lower panel). (**D**) LC-MS/MS showed that free ζ-globin chains were detectable only in αEEP-positive patients and absent in αEEP-negative patients, with no difference observed in other free globin chains. (**E**) Cost analysis showed that the αEEP method was 98.6% and 97.3% less expensive than HbHi and ICT, respectively

The characteristics of the 820 patients included are summarised in Table 1. The cohort consisted predominantly of adults aged 18 years or older (691 patients, 84.3%), and most were Chinese (776 patients, 94.6%). 362 (44.1%) carried one or more α-thalassaemia mutations: α^0^-thalassaemia trait (263, 32.1%); heterozygous α^+^-thalassaemia (97, 11.8%); and homozygous or compound heterozygous α^+^-thalassaemia (2, 0.2%) (Fig. 2A). Among the 263 α^0^-thalassaemia mutations detected, most (259, 98.5%) were --^SEA^ and 4 (1.5%) were --^FIL^. Furthermore, 186 (22.7%) patients were β-thalassaemia carriers, 3 (0.4%) were δβ-thalassaemia carriers, and 11 (1.3%) had heterozygous or homozygous Hb E. 287 (35.0%) patients had no thalassaemia or haemoglobinopathy detected.

**Table 1:** Clinical characteristics of the patients included in the multicentre evaluation

Total no. of cases	820
Age (year) - median (range)	43 (30–63)
-1 to < 18	129 (15.7%)
-18 or above	691 (84.3%)
Female sex - no. (%)	472 (57.6%)
Chinese ethnicity - no. (%)	776 (94.6%)
RBC (×10^12^/L) - median (IQR)	5.06 (4.54–5.61)
HGB (g/dL) - median (IQR)	11.5 (10.3–12.7)
MCV (fL) - median (IQR)	71 (65–78)
MCH (pg) - median (IQR)	22.2 (20.5–25.3)
MCHC (pg) - median (IQR)	31.9 (31.1–32.7)
HCT (L/L) - median (IQR)	0.36 (0.324–0.397)
RDW (%) - median (IQR)	15.6 (14.5–17)
α-thalassaemia - no. (%)	
-Heterozygous α^0^-thalassaemia --^SEA^ mutation	259 (31.6%)
-Heterozygous α^0^-thalassaemia --^FIL^ mutation	4 (0.5%)^*^
-Heterozygous or compound heterozygous α^+^-thalassaemia mutations	2 (0.2%)
-Heterozygous α^+^-thalassaemia mutations	97 (11.8%)
β-thalassaemia, δβ-thalassaemia or Hb E - no. (%)	
-β-thalassaemia trait	186 (22.7%)
-δβ-thalassaemia trait	3 (0.4%)
-Hb E trait	9 (1.1%)
-Homozygous Hb E	1 (0.1%)
-Hb E trait + Hb Kaohsiung	1 (0.1%)
Other non-thalassaemic Hb variants - no. (%)	
-Hb S trait	1 (0.1%)
-Hb G-Taipei	1 (0.1%)
-Hb J-Kaohsiung	1 (0.1%)
-Hb Hekinan II	1 (0.1%)
Hb F level - no. (%)	
-<1%	633 (77.2%)
-≥1%	187 (22.8%)

Abbreviations: --^*FIL*^ Filipino deletion, *Hb* haemoglobin, *HGB* haemoglobin level, *HCT* haematocrit, *MCH* mean cell haemoglobin, *MCHC* mean cell haemoglobin concentration, *MCV* mean cell volume, *RBC* red blood cell count, *RDW* red cell distribution width, --^*SEA*^ Southeast Asian deletion

^*^Extended genotyping performed only in non-Chinese patients

#### Test performance in detecting different α-globin genotypes

The αEEP, HbHi and ICT results are compared against the α-globin genotype in Table 2. The αEEP was 99.6% sensitive (258/259) and 100% specific (561/561) for --^SEA^ carriers. The αEEP was negative in all four non-Chinese --^FIL^ carriers and was equivocal (analysed as negative in the statistical analysis) in one --^SEA^ carrier (Supplementary Fig. S1). The αEEP showed superior diagnostic performance compared with both HbHi (sensitivity 95.8% [*P* = 0.006], specificity 97.3% [*P* < 0.001]) and ICT (sensitivity 95.8% [*P* = 0.006], specificity 75.4% [*P* < 0.001]) for detecting --^SEA^ (Fig. 2B, Supplementary Table S1). All the αEEP, HbHi and ICT were insensitive for α^+^-thalassaemias (0%, 4.0% and 40.4%, respectively) (Supplementary Table S2).

**Table 2:** Summary of the αEEP, HbHi and ICT for α-thalassaemia according to the genotype

α-β Genotypes	αEEP			HbHi		ICT		
	Positive	Equivocal	Negative	Occasional	Negative	Positive	Weak Positive	Negative
α^0^-thalassaemia trait (*N* = 263)								
--^SEA^/α^Hekinan II^α; β^A^β^A^	1	0	0	1	0	1	0	0
--^SEA^/αα; β^A^β^A^	245	1	0	240	6	162	78	6
--^SEA^/αα; β^0^β^A^	11	0	0	6	5	1	5	5
--^SEA^/αα; δβ^0^β^A^	1	0	0	1	0	1	0	0
--^FIL^/αα; β^A^β^A^	0	0	2	2	0	1	1	0
--^FIL^/αα; β^0^β^A^	0	0	1	0	1	0	1	0
--^FIL^/αα; β^J−Kaohsiung^β^A^	0	0	1	1	0	1	0	0
Homozygous or compound heterozygous α^+^-thalassaemia (*N* = 2)								
α^CS^α/α^QS^α; β^A^β^A^	0	0	1	0	1	1	0	0
-α^3.7^/-α^3.7^; β^0^β^A^	0	0	1	0	1	0	0	1
Heterozygous α^+^-thalassaemia (*N* = 97)								
-α^3.7^/αα; β^A^β^A^	0	0	48	0	48	1	14	33
-α^4.2^/αα; β^A^β^A^	0	0	15	1	14	1	7	7
-α^3.7^/αα; β^S^β^A^	0	0	1	0	1	0	0	1
-α^3.7^/αα; β^0^β^A^	0	0	8	0	8	1	1	6
-α^4.2^/αα; β^0^β^A^	0	0	2	0	2	0	0	2
-α^3.7^/αα; β^E^β^Kaohsiung^	0	0	1	0	1	0	0	1
-α^4.2^/αα; β^E^β^A^	0	0	1	0	1	0	0	1
-α^3.7^/αα; δβ^0^β^A^	0	0	1	0	1	0	1	0
α^Codon30^α/αα; β^A^β^A^	0	0	2	1	1	1	1	0
α^CS^α/αα; β^A^β^A^	0	0	3	0	3	0	2	1
α^QS^α/αα; β^A^β^A^	0	0	12	2	10	1	7	4
α^CS^α/αα; β^0^β^A^	0	0	1	0	1	1	0	0
α^QS^α/αα; β^0^β^A^	0	0	2	0	2	0	0	2
Normal α-globin genotype (*N* = 458)								
αα/αα; β^A^β^A^	0	0	287	6	281	4	49	234
αα/αα; β^G−Taipei^β^A^	0	0	1	0	1	0	1	0
αα/αα; β^0^β^A^	0	0	160	2	158	4	34	122
αα/αα; β^E^β^A^	0	0	8	0	8	0	1	7
αα/αα; β^E^β^E^	0	0	1	0	1	0	0	1
αα/αα; δβ^0^β^A^	0	0	1	0	1	0	1	0
Subtotal	258	1	561	263	557	182	204	434
Total	820			820		820		

Abbreviations: *αEEP* α-thalassaemia early eluting peak, *CS* Constant Spring, --^*FIL*^ Filipino deletion, *HbHi* haemoglobin H inclusion test, *ICT* immunochromatographic strip test, *QS* Quong Sze, --^*SEA*^ Southeast Asian deletion

#### Subgroup analyses for --^SEA^ detection according to β-thalassaemia status and Hb F levels

Subgroup analyses for the test performance in detecting --^SEA^ stratified by β-thalassaemia status and Hb F levels are summarised in Supplementary Tables S3 and S4.

Notably, sensitivity was significantly lower in β-thalassaemia carriers than in non-carriers for both HbHi (54.6% vs. 97.6%, *P* < 0.001) and ICT (54.6% vs. 97.6%, *P* < 0.001). Among the eleven patients with concomitant --^SEA^ and β-thalassaemia trait, four were negative for both HbHi and ICT, one was HbHi-positive/ICT-negative, and one was HbHi-negative/ICT-weak positive. All were αEEP-positive. The PPV for ICT was also significantly lower in the β-thalassaemia subgroup (12.5% vs. 71.6%, *P* < 0.001). In contrast, αEEP performance was unaffected by β-thalassaemia status (β-thalassaemia carriers vs. non-carriers: sensitivity 100% vs. 99.6% [*P* = 1]; specificity 100% vs. 100% [*P* = 1]; PPV 100% vs. 100% [*P* = 1]; NPV 100% vs. 99.7% [*P* = 1]) (Supplementary Table S4).

In patients with Hb F ≥ 1%, ICT showed significantly lower specificity (68.6% vs. 78.0%, *P* = 0.02) and PPV (38.8% vs. 70.9%, *P* < 0.001) compared with patients with Hb F < 1%. On the other hand, HbHi had reduced PPV when Hb F ≥ 1% compared with < 1% (83.3% vs. 96.0%, *P* = 0.009). The αEEP remained unaffected by the Hb F levels (Hb F ≥ 1% vs. Hb F < 1%: sensitivity 100% vs. 99.6% [*P* = 1]; specificity 100% vs. 100% [*P* = 1]; PPV 100% vs. 100% [*P* = 1]; NPV 100% vs. 99.8% [*P* = 1]) (Supplementary Table S4).

#### LC-MS/MS study results

Upon database search for globin chain peptides detected in LC-MS/MS, unique peptide sequences for the ζ-globin chain were found in all ten αEEP-positive patients (Fig. 2C). In contrast, unique ζ-globin peptide sequences were absent in all ten αEEP-negative cases (*P* < 0.001; Fig. 2D, Supplementary Table S5). No significant differences were observed in other free globin chains. These findings suggest that the αEEP likely corresponds to ζ-globin chains.

#### Cost analysis

Among the three tests, HbHi incurred the highest cost, while the αEEP had the lowest cost. The αEEP required only 6 s per case for interpretation and recording of the results, with no additional reagent/consumable cost. ICT had the highest reagent/consumable costs. Overall, the αEEP offered a cost reduction of 98.6% and 97.3% compared with HbHi and ICT, respectively (Fig. 2E, Supplementary Table S6).

## Discussion

Thalassaemia screening is important in prevalent regions for identifying potential carriers. Testing is typically requested during investigations for hypochromic microcytic anaemia or as part of the antenatal screening. While carriers are generally asymptomatic, reliable screening is crucial for identifying pregnancies at risk of having offspring with lifelong transfusion dependence or fatal hydrops foetalis [3, 21].

Current antenatal thalassaemia screening guidelines indicate that existing α-thalassaemia screening tests are unreliable, necessitating genotyping for many patients with low mean cell haemoglobin or mean cell volume [9, 10]. This study addresses this challenge by enabling a novel “all-in-one” HPLC screening strategy for --^SEA^ carriers, β-thalassaemia and haemoglobinopathies through the additional analysis of the previously unassessed early eluting peaks.

The sensitivity of the αEEP for detecting --^SEA^ in this study was significantly higher than initially reported [17]. This discrepancy is attributed to limited, non-random sampling for molecular confirmation in the previous study, which overrepresented discordant cases. In contrast, our study evaluated diagnostic performance more accurately, with full genotyping results available in all included cases.

Interestingly, one (0.1%) equivocal αEEP result was observed (Table 1; Supplementary Fig. S1). We adopted a conservative approach due to the potential inter-observer variability, as previously reported [18], resulting in the only false-negative αEEP result. However, an equivocal result should prompt further investigation with genotyping, as it may indicate a hidden αEEP (Supplementary Fig. S1). Further optimisation of the HPLC protocol to better separate the αEEP from other early eluting peaks may also improve the diagnostic performance.

This study revealed a finding of reduced ICT sensitivity in β-thalassaemia carriers, which has not been reported previously. The reduced HbHi sensitivity in β-thalassaemia is well established, as the ameliorated α-/β-globin chain imbalance leads to reduced Hb H (β_4_) formation [22]. Similarly, β-thalassaemia could result in fewer free γ-globin chains available for Hb Bart’s (γ_4_) formation, thereby reducing ICT sensitivity. This phenomenon was unrecognised in previous studies due to a lack of concomitant α- and β-thalassaemia cases [16, 23]. Additionally, ICT demonstrated significantly reduced specificity when Hb F was ≥ 1% compared with < 1% (68.6% vs. 78.0%, *P* = 0.02). Low ICT specificity (45.0%) has been reported in clinical samples with Hb F ≥ 2%, although no comparison arm was available [18]. Our findings also support that ICT is susceptible to interference by elevated Hb F levels, attributable to increased free γ-globin chains and consequently Hb Bart’s formation [18].

In contrast, the αEEP remained accurate in the presence of β-thalassaemia and elevated Hb F levels, likely due to its nature being the embryonic ζ-globin chain confirmed by LC-MS/MS. Although the ζ-globin gene is normally silenced during the developmental globin switch, it is de-repressed in --^SEA^ carriers due to α-globin gene deletion and potential regulatory sequences *in cis* [24, 25]. As ζ-globin is more stable as monomer than as dimer (ζβ) or tetramer (Hb Portland II, ζ_2_β_2_), it is detectable within the early phase of HPLC (containing free globin chains) as an additional early eluting peak (i.e. the αEEP) [26, 27]. Therefore, the αEEP detects --^SEA^ independent of the α-/β-globin imbalance, leading to robustness in cases with β-thalassaemia and elevated Hb F levels. Furthermore, four non-Chinese --^FIL^ carriers were identified, all of whom showed negative αEEP results. This confirms our LC-MS/MS results, as the --^FIL^ mutation involves ζ-globin gene deletion and lacks detectable ζ-globin chains [13]. Collectively, the αEEP is more accurately described as the “free ζ-globin (fζ) peak” to reflect its underlying principle of α-thalassaemia detection.

Our results have significant implications for the αEEP applicability in other ethnicities. The αEEP will likely be highly effective in most Southeast Asia and West Pacific countries where --^SEA^ is the single prevalent double gene deletion, including China, Thailand, Vietnam and Malaysia [5–8, 28]. The αEEP will also be beneficial in countries where other double gene deletions are prevalent in addition to --^SEA^, such as the Philippines [29]. The αEEP is likely to have limited utility in regions where --^SEA^ is uncommon. As illustrated in this study, the importance of ethnicity information cannot be overemphasised to provide appropriate screening and genetic counselling [9].

The characteristics of the αEEP, HbHi and ICT are compared in Table 3. All three methods are based on distinct mechanisms of α-thalassaemia detection: αEEP detects free ζ-globin chains, HbHi detects Hb H (β_4_), and ICT detects Hb Bart’s (γ_4_). Overall, αEEP offers the best diagnostic performance in detecting --^SEA^, demonstrating robustness in the presence of β-thalassaemia and elevated Hb F levels, alongside significant cost reduction. The receiver operating characteristic curve analysis also showed that the αEEP has the greatest area under curve, and both ‘equivocal’ and ‘positive’ results were found to be predictive for --^SEA^ mutation (Supplementary Fig. S2; Supplementary Table S7). Nevertheless, any equivocal αEEP result should be further investigated with additional confirmatory test due to potential inter-observer variation and limited number of cases encountered in this study. Furthermore, αEEP was shown to be suitable for clinical laboratory use, as it is stable, robust against other clinical interferences, and can be easily trained with high inter-observer agreement [18]. The perfect inter-observer agreement of the αEEP was also confirmed in this study (κ = 1.000) (Supplementary Table S8). This method is also highly scalable, potentially also to resource-limited settings, as HPLC equipment is widely used for thalassaemia and haemoglobinopathy screening. Based on our findings, a comprehensive thalassaemia screening flowchart incorporating the αEEP was proposed (Supplementary Fig. S3).

**Table 3:** Summary for the comparison of the αEEP, HbHi and ICT tests

Test	αEEP (or fζ peak)	HbHi	ICT
Testing principle	Free embryonic ζ-globin chain	Hb H (β_4_ tetramers)	Hb Bart’s (γ_4_ tetramers)
--^SEA^ detection	99.6% sensitivity 100% specificity	95.8% sensitivity 97.3% specificity	95.8% sensitivity 75.4% specificity
Effect of β-thalassaemia	Not affected	Reduced sensitivity (reduced α-/β-globin chain imbalance)	Reduced sensitivity (reduced α-/β-globin chain imbalance)
Effect of elevated Hb F level	Not affected	Not affected	Reduced specificity (increased γ-globin production)
Applicability to other double gene deletions	Limited	Applicable	Applicable
Manpower	6 s per case	Up to 15 min per case	3 min per case
Consumables	No additional cost (if HPLC is used already)	USD 12.8 per 100 tests	USD 455.1 per 100 tests
Total cost	Minimal	High manpower cost	High consumable cost

Abbreviations: *αEEP* α-thalassaemia early eluting peak, *fζ* free ζ-globin, *Hb* haemoglobin, *HbHi* haemoglobin H inclusion test, *HPLC* high-performance liquid chromatography, *ICT* immunochromatographic strip test, --^*SEA*^ Southeast Asian deletion

There are several key strengths of our study. First, it is the largest study to date in which all cases underwent confirmatory genotyping, enabling a head-to-head comparison of newer α-thalassaemia screening tests with impartial assessment by blinded observers. Second, the unbiased inclusion of cases from service laboratories ensured real-world applicability. Notably, Hb H disease cases were excluded from this study as they show a characteristic Hb H peak on HPLC and are easily detected by HbHi. This was to avoid overestimating the tests’ ability to detect the asymptomatic α^0^-thalassaemia carriers, which was the primary outcome of interest. Third, we elucidated the previously unknown nature of the αEEP, providing a scientific basis for its use as a reliable diagnostic marker. Given its perfect specificity in our cohort, a positive αEEP result is virtually diagnostic of --^SEA^ carrier status. This finding suggests predictable applicability in other ethnic populations. Lastly, our study offers a practical one-stop solution for screening --^SEA^, β-thalassaemia and haemoglobinopathy carriers by repurposing existing HPLC equipment, thereby streamlining the process. A clear negative αEEP result makes --^SEA^ highly unlikely, overcoming the historical lack of a reliable screening test.

The primary limitation is that this study was conducted in a predominantly Chinese population. Although the applicability of the αEEP is predictable given the well-documented prevalence of α-thalassaemia mutations globally, it is prudent to validate its diagnostic performance in specific regional settings. Since the genotyping study was a targeted panel on common mutations, rare mutations could be missed by the genotyping. Some mutations prevalent in other regions (e.g. --^MED^ and --^THAI^ mutations) were not encountered in our study. While --^THAI^ mutation involves ζ-globin gene deletion and is mechanistically less likely to be αEEP-positive, --^MED^ mutation has intact ζ-globin gene *in cis*. However, the diagnostic performance of the αEEP in these situations should be confirmed in real patients. In addition, while subgroup analyses suggested that the diagnostic performance of αEEP remained robust, ICT and HbHi were significantly affected by concomitant β-thalassaemia or elevated Hb F levels. However, the number of cases with concomitant --^SEA^ mutations was limited (Supplementary Table S3), leading to wider 95% confidence intervals in sensitivity and PPV calculations (Supplementary Table S4). A larger cohort of patients with these conditions may be needed to better confirm diagnostic performance in these settings. Finally, the applicability of the αEEP in neonates and infants are yet to be validated.

## Conclusion

Overall, the αEEP, or free ζ-globin (fζ) peak, is a highly reliable diagnostic marker for --^SEA^ carriers showing superior diagnostic performance compared with existing methods including ICT and microscopy. It enables an “all-in-one” HPLC screening strategy for --^SEA^, β-thalassaemia and other haemoglobinopathies. This method can be readily evaluated and implemented in clinical laboratories, as the required HPLC instrument is widely available.

## Supplementary Information

Below is the link to the electronic supplementary material.

Supplementary Material 1 (DOCX 850 KB)

## Data Availability

The authors confirm that the data supporting the findings of this study are available within the article and/or its supplemental data.
