# Supplementary material for: Alpha-thalassaemia early eluting peak for alpha-thalassaemia --SEA carrier screening: a multicentre diagnostic comparison with immunochromatographic strip test and haemoglobin H inclusion test
Source: Ann Hematol. 2026 Jun 19;105(9):409. doi: 10.1007/s00277-026-07102-0 (PMC13547154; doi:10.1007/s00277-026-07102-0)
Supplement: Supplementary file 1 [file 277_2026_7102_MOESM1_ESM.docx]

**Title: Alpha-thalassaemia early eluting peak for alpha-thalassaemia --^SEA^ carrier screening: a multicentre diagnostic comparison with immunochromatographic strip test and haemoglobin H inclusion test**

**SUPPLEMENTARY INFORMATION**

Page

**Supplementary Methods**

Multicentre diagnostic test comparison	3

Liquid chromatography-tandem mass spectrometry analysis	5

**Supplementary Results**

Supplementary Figure

Supplementary Figure S1. The equivocal α-thalassaemia	7

early eluting peak result

Supplementary Figure S2. The receiver operating characteristic	8
curve of the α-thalassaemia early eluting peak (αEEP),
haemoglobin H inclusion test (HbHi) and
immunochromatographic strip test (ICT) results.

Supplementary Figure S3. Proposed comprehensive	9
thalassaemia screening incorporating the α-thalassaemia early
eluting peak assessment.

Supplementary Tables

Supplementary Table S1. Test performance of the	10

α-thalassaemia screening tests in detecting --^SEA^ mutation.

Supplementary Table S2. Test performance of the	11

α-thalassaemia screening tests in detecting α^+^-thalassaemias.

Supplementary Table S3. The truth tables of the αEEP, ICT	12

and HbHi according to the β-thalassaemia status and the Hb F

level.

Supplementary Table S4. Subgroup analyses for --^SEA^	13

detection according to the β-thalassaemia status and the Hb F

level.

Supplementary Table S5. The LC-MS/MS results according	14

to the αEEP status.

Supplementary Table S6. Cost analysis of the three	15

α-thalassaemia screening tests.

Supplementary Table S7. The area under the receiver	16
operating characteristic curve of the three α-thalassaemia
screening tests for detecting --^SEA^ mutation.

Supplementary Table S8. Inter-observer agreement results	17
for the α-thalassaemia early eluting peak.

**References**	18

# Appendix. Training materials for α-thalassaemia early eluting 19 peak interpretation

**Supplementary Methods**

**Multicentre diagnostic test comparison**

Red cell indices, haemoglobin pattern study and assessment of early eluting peak pattern

Red cell indices were determined by the automatic blood cell counters in the participating laboratories. The haemoglobin (Hb) pattern study included blinded assessment of Hb H inclusion test, automated Variant II high-performance liquid chromatography (HPLC) analysis (β-thalassaemia Short Program, Bio-Rad Laboratories, California, USA) and the assessment of αEEP in the HPLC software as previously described.^1^ The observers for the αEEP were trained for the interpretation of early eluting peak quality and αEEP results. The result of the αEEP was interpreted qualitatively as ‘positive’, ‘equivocal’ or ‘negative’. Equivocal results were recorded but were analysed as ‘negative’ during statistical analysis due to potential inter-observer variability, as previously described.^2^ Training materials distributed to the participating centres were included in the Appendix of this document.

Immunochromatographic strip test

The commercially available iLab αTHAL immunochromatographic strip test kit (i+Med Laboratories, Thailand) based on detection of Hb Bart’s was used in the participating laboratories. The procedure was performed as previously described.^2^ Briefly, one part of the EDTA blood sample was mixed with one part of the lysis buffer using a vortex mixer, followed by vertical immersion in the hemolysed blood for five minutes, avoiding contact with the wall of the test tube. Afterwards, the removed strip was washed with wash buffer provided until the background was clear. The result was interpreted within ten minutes. The control line should be visible for a valid result. The ICT test line was read qualitatively as ‘positive’, ‘weak positive’ or ‘negative’ as previously described.^3^

Molecular study for α-globin genotype

For the α-globin genotyping, genomic DNA was extracted from the EDTA blood sample. All cases were studied for the α-globin genotype targeting the common mutations in the Chinese population based on polymerase chain reaction (PCR) using the previously described protocol or another clinically validated alternative method.^1^ The consensus genotyping panel analysed the six common mutations in Chinese: Southeast Asian type deletion (--^SEA^), 3.7kb deletion (-α^3.7^), 4.2 kb deletion (-α^4.2^), Hb Constant Spring mutation, Hb Quong Sze mutation and codon 30 mutation.

Extended genotyping panel for non-Chinese patients

Extended genotyping by a multiplex gap PCR modified from Tan et al.^4^ targeting the -α^3.7^, --^SEA^, Thai (--^THAI^), Mediterranean (--^MED^) and Filipino (--^FIL^) deletions was performed. Briefly, the extracted genomic DNA was amplified in the ProFlex PCR System (Applied Biosystems, Massachusetts) using the following cycling parameters: initial denaturation at 96°C for 15 min, followed by 30 cycles of 98°C denaturing for 45 s, 60°C annealing for 1 min 30 s, and 72°C extension 2 min 15 s. A final 5 min extension completed the reaction. Each PCR was set up in a total volume of 50 μL containing approximately 300 ng genomic DNA, 0.2 mM of each dNTP, 1.5 mM MgCl2, 1X Q-solution (Qiagen, Germany), 2.5 U HotStarTaq DNA polymerase in supplied reaction buffer (Qiagen) and 13 different primers at various concentrations (see below). Five μL of each amplified product was analysed by electrophoresis on a 1% agarose gel after staining with GelRed nucleic acid gel stain.

| Primer sequences for α-thalassaemia multiplex gap PCR for detection of 3.7 kb, --^SEA^, --^THAI^, --^MED^ and --^FIL^ deletions | | | | |
| --- | --- | --- | --- | --- |
| **Allele detected** | **Name** | **Primer sequences (5' → 3')** | **Concentration (μm)** | **Fragment length (bp)** |
| LIS 3'UTR fragment (internal control) | LIS1 (forward) | ATACCATGGTTACCCCATTGAGC | 0.5 | 2350 |
|  | LIS1 (reverse) | AGGGCTCATTACATGTGGACCC | 0.5 |  |
| 3.7 kb deletion | α2/3.7 (forward) | CCCCTCGCCAAGTCCACCC | 0.2 | 2022/2029 |
|  | 3.7 (reverse) | AAAGCACTCTAGGGTCCAGCG | 0.2 |  |
| α2 gene | α2/3.7 (forward) | As above | - | 1800 |
|  | α2 (reverse) | AGACCAGGAAGGGCCGGTG | 0.2 |  |
| --^SEA^ deletion | SEA (forward) | CGATCTGGGCTCTGTGTTCTC | 0.2 | 1349 |
|  | SEA (reverse) | AGCCCACGTTGTGTTCATGGC | 0.2 |  |
| --^THAI^ deletion | THAI (forward) | GACCATTCCTCAGCGTGGGTG | 0.3 | 1153 |
|  | THAI (reverse) | CAAGTGGGCTGAGCCCTTGAG | 0.3 |  |
| --^MED^ deletion | MED (forward) | TACCCTTTGCAAGCACACGTAC | 0.2 | 807 |
|  | MED (reverse) | TCAATCTCCGACAGCTCCGAC | 0.2 |  |
| --^FIL^ deletion | FIL (forward) | TTTAAATGGGCAAAACAGGCCAGG | 1.0 | 546 |
|  | FIL (reverse) | ATAACCTTTATCTGCCACATGTAGC | 1.0 |  |
|  | | | | |

**Liquid chromatography-tandem mass spectrometry (LC-MS/MS) analysis**

Obtaining early eluates from high-performance liquid chromatography (HPLC)

The haemolysates for the LC-MS/MS analysis were prepared by mixing 5 μL of well-mixed EDTA whole blood sample with 1 mL of diluent solution. The haemolysates were centrifuged under 2000 rpm for 10 minutes, followed by the Variant II HPLC analysis (Bio-Rad Laboratories, California) using the β-thalassaemia short program. The eluted fraction within the first minute of the HPLC was manually collected five times in each specimen. A total of about 10 ml of early eluates were obtained from each specimen.

Materials for LC-MS/MS study

Sequencing grade trypsin were purchased from Promega (Wisconsin, USA). Water and acetonitrile (ACN) were purchased from J.T. Baker (Pennsylvania, USA). Triethyl ammonium bicarbonate (TEAB) was purchased from Thermo Fisher (Massachusetts, USA). Formic acid (FA) was purchased from Fisher Scientific (Pennsylvania, USA). All solvents were in LC-MS grade equivalent or higher.

Sample processing

Two rounds of buffer-exchange were performed on the eluates with 25 mM TEAB using the 10 kDa molecular weight cut off (MWCO) filter (Thermo #88517). Protein digestion was then performed by incubation with 20 ng of trypsin overnight at 37°C. Subsequent tryptic peptides were Speedvac dried.

LC-MS/MS analysis

The dried tryptic peptides were subjected to LC-MS analysis. Eluted peptides were analysed with Dionex Ultimate3000 nanoRSLC system (Thermo Scientific, Massachusetts, USA) coupled to Orbitrap Fusion Tribid Lumos mass spectrometer (Thermo Fisher, USA). Peptides were separated on a commercial C18 column (75 μm i.d. × 50 cm length × 2 μm particle size) coupled to a NanoTrap column (75 μm i.d. × 2 cm length × 3 μm particle size) (Thermo Fisher). Separation was attained using a linear gradient of increasing buﬀer B (80% ACN and 0.1% FA) and declining buﬀer (0.1% FA) at 300 nL/min. Buﬀer B was increased to 30% B in 30 min and ramped to 44% B in 6 min followed by a quick ramp to 95% B, where it was held for 3 min before a quick ramp back to 3% B, where it was held and the column was re-equilibrated. Mass spectrometer was operated in positive polarity mode with capillary temperature of 300 °C. Full mass spectrometry (MS) survey scan resolution was set to 30000 with an automatic gain control (AGC) target value of 5 × 10^4^, maximum ion injection time of 50 ms, and for a scan range of 350-1800 m/z. A data-dependent top 10 method was operated during which higher-energy collisional dissociation (HCD) was used. Spectra were obtained at 15000 MS2 resolution with a standard AGC target and maximum ion injection time of 100 ms, 1.6 m/z isolation width, and normalised collisional energy of 31. Preceding precursor ions targeted for HCD were dynamically excluded of 10 s.

Software analysis

Raw mass spectrometry data were processed using PEAKS Studio X+ version 10.5. Raw data were searched against Target Protein database using settings as below: oxidised methionine (M) was selected as dynamic modifications with minimum peptide length of 7 amino acids was enabled. Precursor mass tolerance was within 10 ppm and the fragment ion tolerance was within 0.02 Da, using Orbitrap-Orbitrap and HCD as the instrument settings. Confident criteria -10logp > 20 with false discovery rate (FDR) 1%.

**Supplementary Results**

# Supplementary Figure

Supplementary Figure S1. The equivocal α-thalassaemia early eluting peak result.

The equivocal α-thalassaemia early eluting peak (αEEP) result (left) in comparison with a negative case (upper right) and a positive case (lower right). The equivocal result appears as a slower decline of the peak at 0.20 mins (P_0.20_) with no definite isolated peak appreciable.

Supplementary Figure S2. The receiver operating characteristic curve of the α-thalassaemia early eluting peak (αEEP), haemoglobin H inclusion test (HbHi) and immunochromatographic strip test (ICT) results.

Supplementary Figure S3. Proposed comprehensive thalassaemia screening incorporating the α-thalassaemia early eluting peak assessment.

# Supplementary Tables

| Supplementary Table S1. Test performance of the α-thalassaemia screening tests in detecting --^SEA^ mutation. | | | |
| --- | --- | --- | --- |
| **Test** | **Diagnostic performance - % (95%CI)** | | ***P* value*** |
| **--^SEA^ (N=259)** | | | |
| αEEP | Sensitivity | 99.6% (97.9%-100.0%) |  |
|  | Specificity | 100% (99.3%-100.0%) |  |
|  | PPV | 100% (98.6%-100.0%) |  |
|  | NPV | 99.8% (99.0%-100.0%) |  |
| HbHi | Sensitivity | 95.8% (92.6%-97.6%) | 0.006 |
|  | Specificity | 97.3% (95.6%-98.4%) | <0.001 |
|  | PPV | 94.3% (90.8%-96.5%) |  |
|  | NPV | 98.0% (96.5%-98.9%) |  |
| ICT | Sensitivity | 95.8% (92.6%-97.6%) | 0.006 |
|  | Specificity | 75.4% (71.7%-78.9%) | <0.001 |
|  | PPV | 64.3% (59.4%-68.9%) |  |
|  | NPV | 97.5% (95.5%-98.6%) |  |
| *Compared with the αEEP by McNemar's test. | | | |
| Abbreviations: αEEP, α-thalassaemia early eluting peak; HbHi, haemoglobin H inclusion test; ICT, immunochromatographic strip test; NPV, negative predictive value; PPV, positive predictive value; --^SEA^, Southeast Asian deletion. | | | |

| Supplementary Table S2. Test performance of the α-thalassaemia screening tests in detecting α^+^-thalassaemias. | | |
| --- | --- | --- |
| **Test** | **Diagnostic performance - % (95%CI)** | |
| **α^+^-thalassaemias (N=99)** | | |
| αEEP | Sensitivity | 0.0% (0.0%-3.7%) |
|  | Specificity | 64.2% (60.7%-67.6%) |
|  | PPV | 0.0% (0.0%-1.5%) |
|  | NPV | 82.4% (79.0%-85.3%) |
| HbHi | Sensitivity | 4.0% (1.6%-9.9%) |
|  | Specificity | 64.1% (60.5%-67.5%) |
|  | PPV | 1.5% (0.6%-3.8%) |
|  | NPV | 82.9% (79.6%-85.8%) |
| ICT | Sensitivity | 40.4% (31.3%-50.3%) |
|  | Specificity | 52.0% (48.4%-55.6%) |
|  | PPV | 10.4% (7.7%-13.8%) |
|  | NPV | 86.4% (82.9%-89.3%) |
| Abbreviations: αEEP, α-thalassaemia early eluting peak; HbHi, haemoglobin H inclusion test; ICT, immunochromatographic strip test; NPV, negative predictive value; PPV, positive predictive value. | | |

| Supplementary Table S3. The truth tables of the αEEP, ICT and HbHi according to the β-thalassaemia status and the Hb F level. | | | | | |
| --- | --- | --- | --- | --- | --- |
|  | | **β-thalassaemia status** | | | |
|  |  | **β-thalassaemia (N=186)** | | **No β-thalassaemia (N=634)** | |
| --^SEA^ mutation | | Present (N=11) | Absent (N=175) | Present (N=248) | Absent (N=386) |
| αEEP | Positive | 11 | 0 | 247 | 0 |
|  | Equivocal | 0 | 0 | 1 | 0 |
|  | Negative | 0 | 175 | 0 | 386 |
| HbHi | Occasional | 6 | 2 | 242 | 13 |
|  | Negative | 5 | 173 | 6 | 373 |
| ICT | Positive | 1 | 6 | 164 | 11 |
|  | Weak Positive | 5 | 36 | 78 | 85 |
|  | Negative | 5 | 133 | 6 | 290 |
|  | | **Hb F level** | | | |
|  |  | **≥1% (N=187)** | | **<1% (N=633)** | |
| --^SEA^ mutation | | Present (N=31) | Absent (N=156) | Present (N=228) | Absent (N=405) |
| αEEP | Positive | 31 | 0 | 227 | 0 |
|  | Equivocal | 0 | 0 | 1 | 0 |
|  | Negative | 0 | 156 | 0 | 405 |
| HbHi | Occasional | 30 | 6 | 218 | 9 |
|  | Negative | 1 | 150 | 10 | 396 |
| ICT | Positive | 28 | 9 | 137 | 8 |
|  | Weak Positive | 3 | 40 | 80 | 81 |
|  | Negative | 0 | 107 | 11 | 316 |
| Abbreviations: αEEP, α-thalassaemia early eluting peak; Hb, haemoglobin; HbHi, haemoglobin H inclusion test; ICT, immunochromatographic strip test; --^SEA^, Southeast Asian deletion. | | | | | |

| Supplementary Table S4. Subgroup analyses for --^SEA^ detection according to the β-thalassaemia status and the Hb F level. | | | | |
| --- | --- | --- | --- | --- |
| **Test** | **Diagnostic performance - % (95% CI)** | **β-thalassaemia status** | | ***P* value** |
|  |  | **β-thalassaemia** | **No β-thalassaemia** |  |
| αEEP | Sensitivity | 100% (74.1%-100.0%) | 99.6% (97.8%-100.0%) | 1 |
|  | Specificity | 100% (97.9%-100.0%) | 100% (99.0%-100.0%) | 1 |
|  | PPV | 100% (74.1%-100.0%) | 100% (98.5%-100.0%) | 1 |
|  | NPV | 100% (97.9%-100.0%) | 99.7% (98.6-100.0%) | 1 |
| HbHi | Sensitivity | 54.6% (28.0%-78.7%) | 97.6% (94.8%-98.9%) | <0.001 |
|  | Specificity | 98.9% (95.9%-99.8%) | 96.6% (94.3%-98.0%) | 0.16 |
|  | PPV | 75.0% (40.9%-95.6%) | 94.9% (91.4%-97.0%) | 0.07 |
|  | NPV | 97.2% (93.6%-98.8%) | 98.4% (96.6%-99.3%) | 0.34 |
| ICT | Sensitivity | 54.6% (28.0%-78.7%) | 97.6% (94.8%-98.9%) | <0.001 |
|  | Specificity | 76.0% (69.2%-81.7%) | 75.1% (70.6%-79.2%) | 0.92 |
|  | PPV | 12.5% (5.9%-24.7%) | 71.6% (66.6%-76.1%) | <0.001 |
|  | NPV | 96.4% (91.8%-98.4%) | 98.0% (95.7%-99.1%) | 0.34 |
| **Test** | **Diagnostic performance - % (95% CI)** | **Hb F level** | | ***P* value** |
|  |  | **≥1%** | **<1%** |  |
| αEEP | Sensitivity | 100% (89.0-100.0%) | 99.6% (97.6%-100.0%) | 1 |
|  | Specificity | 100% (97.6%-100.0%) | 100% (99.1%-100.0%) | 1 |
|  | PPV | 100% (89.0%-100.0%) | 100% (98.3%-100.0%) | 1 |
|  | NPV | 100% (97.6%-100.0%) | 99.8% (98.6-100.0%) | 1 |
| HbHi | Sensitivity | 96.8% (83.8-99.8%) | 95.6% (92.1-97.6%) | 1 |
|  | Specificity | 96.2% (91.9%-98.2%) | 97.8% (95.8%-98.8%) | 0.38 |
|  | PPV | 83.3% (68.1%-92.1%) | 96.0% (92.6%-97.9%) | 0.009 |
|  | NPV | 99.3% (96.3%-100.0%) | 97.5% (95.5%-98.7%) | 0.3 |
| ICT | Sensitivity | 100% (89.0-100.0%) | 95.2% (91.6%-97.3%) | 0.37 |
|  | Specificity | 68.6% (60.9%-75.4%) | 78.0% (73.7%-81.8%) | 0.02 |
|  | PPV | 38.8% (28.8%-49.7%) | 70.9% (65.6%-75.7%) | <0.001 |
|  | NPV | 100% (96.5%-100.0%) | 96.6% (94.1%-98.1%) | 0.07 |
| Abbreviations: αEEP, α-thalassaemia early eluting peak; Hb, haemoglobin; HbHi, haemoglobin H inclusion test; ICT, immunochromatographic strip test; NPV, negative predictive value; PPV, positive predictive value; --^SEA^, Southeast Asian deletion. | | | | |

| Supplementary Table S5. The LC-MS/MS results according to the αEEP status. | | | | |
| --- | --- | --- | --- | --- |
| Proteins (unique peptide sequences) | LC-MS/MS result | αEEP | | *P* value |
|  |  | Positive | Negative |  |
| HBA | Detected | 10 | 10 | 1 |
|  | Not detected | 0 | 0 |  |
| HBB | Detected | 10 | 10 | 1 |
|  | Not detected | 0 | 0 |  |
| HBG2 | Detected | 5 | 7 | 0.65 |
|  | Not detected | 5 | 3 |  |
| HBD | Detected | 7 | 4 | 0.37 |
|  | Not detected | 3 | 6 |  |
| HBAZ | Detected | 10 | 0 | <0.001 |
|  | Not detected | 0 | 10 |  |
| Abbreviations: αEEP, α-thalassaemia early eluting peak; HBA, haemoglobin subunit alpha; HBAZ, haemoglobin subunit zeta; HBB, haemoglobin subunit beta; HBD, haemoglobin subunit delta; HBG2, haemoglobin subunit gamma-2; LC-MS/MS, liquid chromatography-tandem mass spectrometry. | | | | |

| Supplementary Table S6. Cost analysis of the three α-thalassaemia screening tests. | | | | | | | |
| --- | --- | --- | --- | --- | --- | --- | --- |
| Cost (in USD) per 100 tests | Staff operation time | | PE cost of junior technical staff | PE cost of senior technical staff | Reagent / consumable costs | Total cost | Cost reduction by αEEP |
|  | Preparation time | Examination time |  |  |  |  |  |
| HbHi | 300 min (Junior) | 1500 min (Junior) | 1288.5 | 0 | 12.8 | 1301.3 | 98.6% |
| ICT | 300 min (Junior) and 10 min (Senior) | | 214.8 | 11.4 | 455.1 | 681.3 | 97.3% |
| αEEP | 10 min (Junior) and 10 min (Senior) | | 7.2 | 11.4 | 0 | 18.6 |  |
| Abbreviations: αEEP, α-thalassaemia early eluting peak; HbHi, haemoglobin H inclusion test; ICT, immunochromatographic strip test; PE, personnel expenditure. | | | | | | | |

| Supplementary Table S7. The area under the receiver operating characteristic curve of the three α-thalassaemia screening tests for detecting --^SEA^ mutation. | | | |
| --- | --- | --- | --- |
| **Test** | **Best cutoff** | **Area under curve (95% Confidence Interval)** | ***P* value^*^** |
| αEEP | Equivocal | 1.000 (1.000-1.000) | <0.001 |
| HbHi | Occasional | 0.965 (0.949-0.982) | <0.001 |
| ICT | Weak Positive | 0.920 (0.899-0.940) | <0.001 |
| ^*^Null hypothesis: true area = 0.5 | | | |
| Abbreviations: αEEP, α-thalassaemia early eluting peak; HbHi, haemoglobin H inclusion test; ICT, immunochromatographic strip test; --^SEA^, Southeast Asian deletion. | | | |

| Supplementary Table S8. Inter-observer agreement results for the α-thalassaemia early eluting peak. | | | | | |
| --- | --- | --- | --- | --- | --- |
| αEEP result | | Observer 1 | | | Total |
|  |  | Negative | Equivocal | Positive |  |
| Observer 2 | Negative | 561 | 0 | 0 | 561 |
|  | Equivocal | 0 | 1 | 0 | 1 |
|  | Positive | 0 | 0 | 258 | 258 |
| Total | | 561 | 1 | 258 | 820 |
| κ=1.000 [95% CI (bootstrapping with 1,000 samples): 1.000 - 1.000]; *P*<0.001. | | | | | |
| Abbreviation: αEEP, α-thalassaemia early eluting peak; 95% CI, 95% Confidence Interval. | | | | | |

**References**

1. Lam WK, Law WYF, Wong TF, et al. A specific early eluting peak in Bio-Rad Variant II cation-exchange high-performance liquid chromatography (CE-HPLC) for the screening of alpha-thalassaemia in a Chinese population. Pathology. 2023 Oct;55(6):835-842.
2. Lam WK, Fan CPY, Law WYF, et al. Evaluation of stability and potential interference on the α-thalassaemia early eluting peak and immunochromatographic strip test for α-thalassaemia --SEA carrier screening. Clin Chem Lab Med. 2025 Aug 18;63(12):2549-2557.
3. Winichagoon P, Kumpan P, Holmes P, et al. Validation of the immunochromatographic strip for α-thalassemia screening: a multicenter study. Transl Res. 2015 Jun;165(6):689-95.
4. Tan AS, Quah TC, Low PS, Chong SS. A rapid and reliable 7-deletion multiplex polymerase chain reaction assay for alpha-thalassemia. Blood. 2001 Jul 1;98(1):250-1.

**Appendix. Training materials for α-thalassaemia early eluting peak interpretation**

**Recommended area of interest for assessment of αEEP in the chromatogram:**

- Check the “Expand” button in the program
- Y-axis: from about 3.00% to -2.00%
- X-axis: from 0 to 0.70 mins (marking intervals of 0.10 mins)

**Usual early eluting peak pattern**

A satisfactory normal early eluting peaks pattern would contain the following peaks: a void peak (at 0.10 mins), an early eluting peak at 0.20 min (P_0.20_) and altered HbF at 0.50 mins (may not be seen in Hb H disease). Recognising these peaks is important to ensure satisfactory results for interpretation. Repeating with or without washing of red cells may resolve the quality issues.

Adapted from *Pathology*, 55(6), Lam WK, Law WYF, Wong TF, et al., A specific early eluting peak in Bio-Rad Variant II cation-exchange high-performance liquid chromatography (CE-HPLC) for the screening of alpha-thalassaemia in a Chinese population, 835-842, Copyright (2023), with permission from Elsevier.

**Interpretation of αEEP**

(A) The interpretation of αEEP (black arrow) is mostly either ‘negative’ (left panel) or ‘positive’ (right panel). (B) Sometimes the αEEP could be less pronounced but is still obviously ‘positive’. (C) Rarely the αEEP is subtle and appears as shouldering at 0.24 min, and these are interpreted as ‘equivocal’. Further investigations should be performed as these could represent potential --^SEA^ carriers.

Adapted from *Clin Chem Lab Med*, 63(12), Lam WK, Fan CPY, Law WYF, et al., Evaluation of stability and potential interference on the α-thalassaemia early eluting peak and immunochromatographic strip test for α-thalassaemia --^SEA^ carrier screening, 2549-2557, Copyright (2025), with permission from De Gruyter Brill.

αEEP in cases with elevated HbA1c levels

The effects of elevated glycated Hb levels on αEEP. Graphs show the positive correlation between (A) glycated Hb peak (P2) and HbA1c levels and (B) P2 and early eluting peak at 0.20 min (P_0.20_). (C) The P_0.20_ increases with increasing P2 or HbA1c levels. (D) The α-thalassaemia early eluting peak (arrow) remained interpretable in cases with elevated P2 levels.

Reprinted from *Clin Chem Lab Med*, 63(12), Lam WK, Fan CPY, Law WYF, et al., Evaluation of stability and potential interference on the α-thalassaemia early eluting peak and immunochromatographic strip test for α-thalassaemia --^SEA^ carrier screening, 2549-2557, Copyright (2025), with permission from De Gruyter Brill.

αEEP in cases with elevated Hb F levels

The effects of elevated Hb F levels on αEEP. (A) Positive correlation between Hb F and early eluting peak at 0.20 min (P_0.20_). (B) The early eluting peak pattern in cases with different Hb F levels and negative for α-thalassaemia mutations. (C) The α-thalassaemia early eluting peak (arrow) remained interpretable in cases with elevated Hb F levels.

Reprinted from *Clin Chem Lab Med*, 63(12), Lam WK, Fan CPY, Law WYF, et al., Evaluation of stability and potential interference on the α-thalassaemia early eluting peak and immunochromatographic strip test for α-thalassaemia --^SEA^ carrier screening, 2549-2557, Copyright (2025), with permission from De Gruyter Brill.

Hb H disease

This case shows the presence of αEEP (arrow) in both (A) adult and (B) neonatal Hb H diseases, although it is not necessary for the diagnosis of Hb H disease due to the prominent Hb H (white arrowhead) or Hb Bart’s (black arrowhead) peak seen.

Adapted from *Pathology*, 55(6), Lam WK, Law WYF, Wong TF, et al., A specific early eluting peak in Bio-Rad Variant II cation-exchange high-performance liquid chromatography (CE-HPLC) for the screening of alpha-thalassaemia in a Chinese population, 835-842, Copyright (2023), with permission from Elsevier.

**Troubleshooting: suboptimal early eluting peak patterns**

Case 1

The early eluting peaks may rarely show atypical pattern due to problems during the elution process. The altered Hb F peak was not visible in this case (left panel). After repeat, a normal early eluting peak pattern was obtained (right panel) with a negative αEEP result.

Zoom-in images:

Case 2

When the early eluting peaks (void peak and P_0.20_ in this case) are abnormally high with a normal Hb F, there is likely a quality issue and a repeat could help resolve the issue.

Zoom-in images:

**Quiz for training observers**

**Please interpret the following early eluting peak patterns (positive, negative, equivocal, suboptimal).**

Question 1

Positive / Negative / Equivocal / Suboptimal

Question 2

Positive / Negative / Equivocal / Suboptimal

Question 3 (Hb F 10.5%)

 Positive / Negative / Equivocal / Suboptimal

Question 4

Positive / Negative / Equivocal / Suboptimal

Question 5

 Positive / Negative / Equivocal / Suboptimal

Question 6 (Hb F normal)

Positive / Negative / Equivocal / Suboptimal

Question 7

Positive / Negative / Equivocal / Suboptimal

Question 8

 Positive / Negative / Equivocal / Suboptimal

**Answer to Quiz**

Question 1: Negative

Question 2: Suboptimal

Question 3: Positive

Question 4: Negative

Question 5: Positive

Question 6: Suboptimal

Question 7: Positive

Question 8: Negative
